# The Mechanism and Inflammatory Markers Involved in the Potential Use of N-acetylcysteine in Chronic Pain Management

**DOI:** 10.3390/life14111361

**Published:** 2024-10-23

**Authors:** Mona Singh, Alina Kim, Amelie Young, Deanna Nguyen, Cynthia L. Monroe, Tiffany Ding, Dennis Gray, Vishwanath Venketaraman

**Affiliations:** 1 College of Osteopathic Medicine, Western University of Health Sciences, Pomona, CA 91766, USA; mona.singh@westernu.edu (M.S.); alina.kim@westernu.edu (A.K.); amelie.young@westernu.edu (A.Y.); deanna.nguyen@westernu.edu (D.N.); tiffany.ding@westernu.edu (T.D.); 2 College of Medicine, California Northstate University, Elk Grove, CA 95757, USA; cynthia.monroe9780@cnsu.edu; 3 Vigilant Anesthesiology, PA, Tampa, FL 33193, USA; dennisgrayjr@gmail.com

**Keywords:** NAC, neuropathic pain, musculoskeletal pain, opioids, metalloproteinases, ROS, chronic pain

## Abstract

N-acetylcysteine (NAC) has established use as an antidote for acetaminophen overdose and treatment for pulmonary conditions and nephropathy. It plays a role in regulating oxidative stress and interacting with various cytokines including IL-1β, TNFα, IL-8, IL-6, IL-10, and NF-κB p65. The overexpression of reactive oxygen species (ROS) is believed to contribute to chronic pain states by inducing inflammation and accelerating disease progression, favoring pain persistence in neuropathic and musculoskeletal pain conditions. Through a comprehensive review, we aim to explore the mechanisms and inflammatory pathways through which NAC may manage neuropathic and musculoskeletal pain. Evidence suggests NAC can attenuate neuropathic and musculoskeletal pain through mechanisms such as inhibiting matrix metalloproteinases (MMPs), reducing reactive oxygen species (ROS), and enhancing glutamate transport. Additionally, NAC may synergize with opioids and other pain medications, potentially reducing opioid consumption and enhancing overall pain management. Further research is needed to fully elucidate its therapeutic potential and optimize its use in pain management. As an adjuvant therapy, NAC shows potential for chronic pain management, offering significant benefits for public health.

## 1. Introduction

Chronic pain is defined as pain lasting more than 3 months, and the main treatment involves opioids [1]. In addition to the serious consequences on the patient’s life, chronic pain has an exacerbating effect on their social environment, family environment, and on receiving health care services [2]. The use of opioids for the treatment of chronic pain has been currently limited due to the opioid crisis that has led to seeking alternatives such as the use of non-steroidal analgesics or even complementary therapies. The evidence on supplements used as an isolated way to manage pain is not supported, but supplements used as an adjunct may be of benefit. N-acetylcysteine (NAC) has been novel in its use in pain therapy. Historically, NAC has been used as an antidote for acetaminophen overdose, pulmonary conditions, and in certain cases of nephropathy [1]. NAC is involved in regulating oxidative stress, and it is believed that many chronic pain states are associated with an excessive level of reactive oxygen species (ROS) [1]. Under normal physiological conditions, ROS are essential for various cellular processes, including differentiation, proliferation, growth, and apoptosis. However, when present in excess, ROS can inflict damage on cellular structures, including lipids, proteins, and nucleic acids, by elevating oxidative stress [1]. NAC mitigates the damaging effects of ROS by serving as a precursor to the antioxidant glutathione (GSH), which is recognized for its significant antioxidant and anti-inflammatory effects [1]. Oxidative stress is a factor that accelerates disease progression and is believed to favor pain persistence in many chronic inflammatory pain conditions, including musculoskeletal and neuropathic pain. In our comprehensive review, we will be discussing NAC’s primary effects on neuropathic and musculoskeletal pain.

Although it is most known that NAC is used as an acetaminophen antidote, there is limited research on how NAC contributes to reducing chronic pain. In our review, we will specifically and mechanistically describe how NAC reduces neuropathic and musculoskeletal pain, as these systems have been devastating in their effects on chronic pain. First, regarding neuropathic pain, matrix metalloproteinases (MMP)-9 and MMP-2 have previously been described as key components in neuropathic pain because of their facilitation of inflammatory cytokine maturation and induction of neural inflammation [3]. Studies have demonstrated that NAC significantly attenuates neuropathic pain through a unique mechanism of MMP inhibition. Being both an anti-inflammatory and antioxidant agent, NAC also may be used as an adjunct to treat musculoskeletal diseases such as rheumatoid arthritis and inflammatory conditions like osteoarthritis (OA) [4,5]. The unresolved biomechanical and biochemical stress from OA and increased ROS promotes a more dominant catabolic metabolic environment [4]. As a result, the stress creates lesions in subchondral bone and peripheral structures and erosion of cartilage, causing physical pain [5]. In cartilage and synovium derived from human OA patients, NAC may reduce the secretion of PGE2 and the expression of COX-2 and MMP-13 protein in synoviocytes stimulated by IL-1β [4]. It may have a symptomatic effect on the synovium but future studies should observe its structural effect on cartilage [5]. NAC acts to reduce pro-oxidants in inflammatory conditions like neuropathic and musculoskeletal pain, serving to potentially alleviate chronic pain in various disease processes. There are also other musculoskeletal and neuropathic mechanisms involving NAC, which will be discussed in our review.

There is also limited research on NAC’s effects with other pain modalities such as opioids. We will discuss this in our article, which would be beneficial for both post treatment and recovery. One example involves complex regional pain syndrome in rat models. ROS play an important role in the development of complex regional pain syndrome type I (CRPS-I), as also demonstrated with the chronic post ischemia pain (CPIP) animal model of CRPS-I [6]. An animal model study showed that morphine and the antioxidant NAC act synergistically to reduce mechanical allodynia in CPIP rats [6]. Other pilot trials have shown that patients took less opioids following spine surgery when taken with NAC as well as reported less pain [7]. This shows that NAC can be used as an adjunct for complex regional pain syndrome and postoperative conditions, which would be another condition to be managed thoroughly using NAC as an adjunct, which we will elaborate on below. Thus, the overall objective in our review is to discuss the mechanisms and inflammatory pathways through which NAC may manage neuropathic and musculoskeletal pain. This is crucial for optimal pain management for patients who suffer from debilitating pain.

## 2. Methodology

Using PubMed and Google Scholar, we conducted a comprehensive review including quantitative and qualitative research, cohort studies, systematic reviews, and randomized control trials mainly between the dates of 2000 and 2024. Keywords in our search included NAC chronic pain, NAC neuropathic pain, NAC musculoskeletal pain, synergy NAC and opioids, n-acetylcysteine, NAC, glutathione, neuropathic pain relief, musculoskeletal pain relief, chronic pain relief, NAC mechanism, analgesia, oxidative stress, reactive oxygen species, pain pathway, opioids, acetaminophen, adjuvant analgesics, NSAIDs, chronic pain management, NAC analgesia neuropathic pain, NAC opioids, NAC analgesia surgery, NAC chronic pain, and NAC analgesia procedure.

## 3. Role of NAC in Oxidative Stress

N-acetylcysteine (NAC) plays a significant role in combating oxidative stress, primarily by facilitating and enhancing glutathione (GSH) synthesis [8]. In accordance with the indirect pathway, NAC reduces oxidative stress by increasing levels of GSH. GSH is an intracellular tripeptide containing cysteine (l-γ-glutamyl–l-cysteinyl–glycine) [8]. As a precursor to the amino acid cysteine, NAC facilitates the production of GSH, the most abundant non-protein thiol in the body and a critical antioxidant in cellular redox homeostasis [8]. GSH not only reacts directly with reactive species but also serves as a cofactor or substrate for various antioxidant enzymes, thus playing a crucial role in cellular defense against oxidative stress [8]. One of the key mechanisms by which NAC exerts its antioxidant effects is by increasing intracellular cysteine levels, which are typically low and thus act as the rate-limiting substrate in GSH biosynthesis [8,9]. Previous studies have shown that using cysteine alone has proven ineffective in raising GSH concentrations, making NAC a pivotal strategy for mitigating oxidative damage, particularly in cases of inflammation associated with GSH deficiency [8]. Furthermore, as an indirect antioxidant, NAC is able to break down thiol proteins, releasing free thiols with higher antioxidant capacity, potentiating GSH biosynthesis and enhancing the overall antioxidant defense system [8,10]. By increasing cysteine levels, NAC increases GSH production as a result and helps serve as an antioxidant and combat oxidative stress.

Furthermore, through its free thiol group and direct pathway, GSH directly scavenges ROS, such as superoxide radicals, hydrogen peroxide (H_2_O_2_), and nitric oxide [11]. For example, hydrogen peroxide, which is highly toxic to membranes, lipids, proteins, and the genome, is reduced and detoxified to water in the reaction H_2_O_2_ + 2GSH → 2H_2_O + GSSG (oxidized glutathione), preventing the harmful and damaging oxidative stress [12]. In its direct pathway neutralizing these free radicals and ROS with its free thiol group, NAC acts as an antioxidant, which can have implications in combatting pain. NAC has also been shown to display chelating action by binding to active redox metal ions such as copper (Cu^2+^), iron (Fe^3+^), and heavy metals like cadmium (Cd^2+^), mercury (Hg^2+^), and lead (Pb^2+^) [8,11,13]. By forming complexes with these ions that are involved in producing oxidative stress, NAC facilitates their excretion from the body and reduces metal toxicity [8]. NAC’s reducing capacity also enables it to restore systemic pools of small thiols and reduce protein sulfhydryl groups involved in regulating the redox state [8]. Even though NAC has the ability to reduce amounts of metal ions in cases of toxicity, studies that observed its chelating properties are limited [8]. It is currently unclear whether NAC acts as a chelator or whether the benefits elicited are mostly related to its action as an indirect antioxidant via an increase in GSH. Despite this, NAC’s involvement in reducing oxidative stress is shown through its indirect and direct pathway of increasing levels of GSH and serving as a scavenger to neutralize ROS. The role it plays in reducing oxidative stress is crucial in its implication to manage chronic pain.

## 4. Culprits in the Pain Pathway and the Impact of NAC

**Figure 1:**
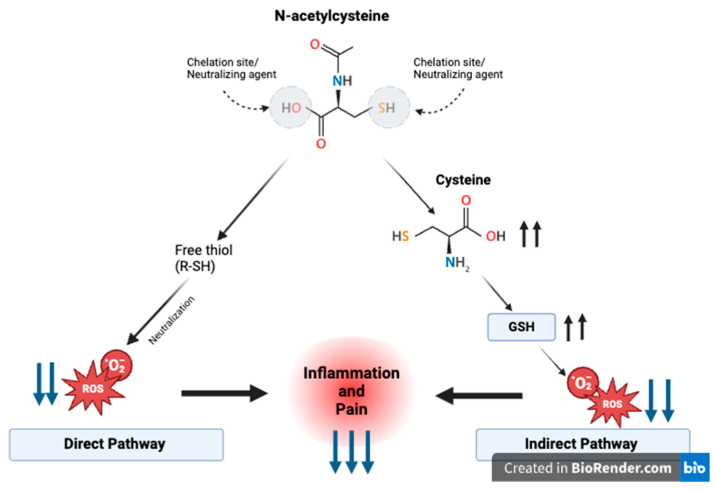
NAC’s role in regulating oxidative stress and reducing ROS, inflammation, and pain. Through the indirect and direct pathways shown sequentially, NAC at the end of the pathways decrease ROS/oxidative stress to decrease inflammation and pain as a result.

**Figure 2:**
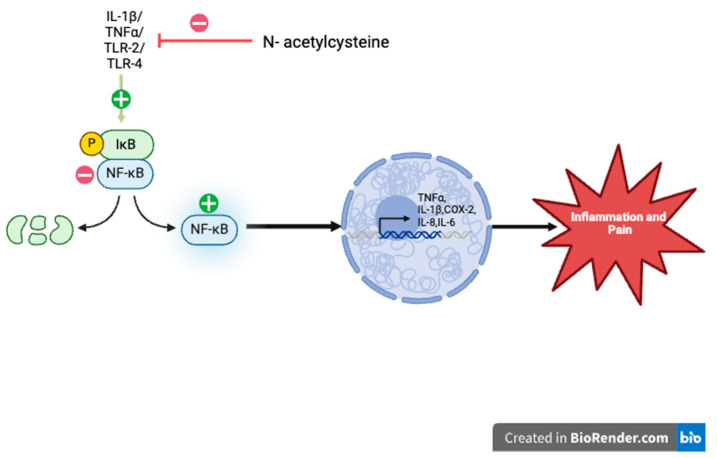
Pain pathway and NAC inhibiting the activation of NF-κB to slow the progression of inflammation and pain. In its inactive state, NF-κB is bound in the cytoplasm to inhibitors of κB (IκBα). Activation by pro-inflammatory cytokines, such as interleukin-1β (IL-1β) and tumor necrosis factor α (TNFα), epidermal growth factors, or CFA-stimulating TLR-2 and TLR-4 leads to IκBα phosphorylation (P symbol ) and its subsequent destruction [14]. This activation of NF-κB (plus sign) through an inflammation state ultimately triggers the transcription of κB-associated inflammatory genes involved in pain indirectly and directly, such as TNFα, IL-1β, and cyclooxygenase-2 (COX-2) (in nucleus in the figure) [14]. NAC inhibits (minus sign) NF-kB activation, thereby suppressing the production of cytokines such as TNFα, IL-1β, IL-8, and IL-6 which are key in the inflammation pathway and progression of chronic pain.

NAC’s antioxidant properties help to act on specific molecular targets of the pain pathway and reduce pain [1,8,14]. During normal physiological conditions, ROS play a role in cellular functions like growth, cell differentiation, and apoptosis [1]. However, in higher concentrations, ROS can cause damage to cellular components by increasing oxidative stress and lead to pain as a result [1]. NAC therefore is generally produced by cellular stress, and since it serves as a precursor of the antioxidant glutathione, it assists with regulating oxidative stress that ultimately contributes to pain [1]. The mechanisms underlying its antioxidative properties are thought to operate via both direct and indirect pathways [1]. The direct mechanism involves the free thiol group of NAC, which interacts with and neutralizes free radicals and ROS [1]. In contrast, the indirect mechanism requires NAC to enter the cell, where it combines with glutamic acid and glycine to increase intracellular levels of glutathione (GSH). It also increases cysteine levels and increases GSH, and this as a result leads to less ROS production [1] (Figure 1). One of NAC’s primary targets in the pain pathway is the nuclear factor kappa-light-chain-enhancer of activated B cells [8]. In its inactive state, NF-κB is bound in the cytoplasm to inhibitors of κB (IκBα). Activation by pro-inflammatory cytokines, such as interleukin-1β (IL-1β) and tumor necrosis factor α (TNFα), epidermal growth factors, or CFA-stimulating TLR-2 and TLR-4 leads to IκBα phosphorylation and its subsequent destruction [14]. This activation through an inflammation state ultimately triggers the transcription of κB-associated inflammatory genes involved in pain indirectly and directly, such as TNFα, IL-1β, and cyclooxygenase-2 (COX-2) [14]. NAC inhibits NF-kB activation, thereby suppressing the production of cytokines such as TNFα, IL-1β, IL-8, and IL-6 which are key in the inflammation pathway and progression of chronic pain [8,15] (Figure 2).

In models of neuropathic pain, such as chronic constriction injury (CCI) of the sciatic nerve in rats, NAC treatment has demonstrated neuroprotective effects [3,16]. Matrix metalloproteinases MMP-9 and MMP-2 play a role in the pain pathway due to their facilitation of cytokine maturation and role in neural inflammation [3]. Li et al.’s experiments of in vivo and in vitro administration of NAC suppressed MMP-9 and MMP-2 activity, attenuating neuropathic pain in rats [3].

NAC’s ROS scavenging abilities in neurons helps to normalize oxidative imbalances altered by ROS such as superoxide radicals, H_2_O_2_, and nitric oxide (NO), which contribute to neuropathic pain [11]. This was demonstrated in rats with CCI, as Horst et al. found NAC treatment increased antioxidant enzyme activity, such as with glutathione-S-transferase and glutathione peroxidase in the spinal cord, and decreased NO metabolism [16]. Lipids can be targeted by ROS to become peroxidized, leading to cellular damage and inflammation. In CCI rodent models, NAC administration has shown to reduce lipid hydroperoxide formation in the spinal cord, further supporting the indication that NAC reduces ROS levels and decreases oxidative stress markers [11]. Moreover, in Sözbir and Nazıroğlu’s studies, NAC showed a protective role in regulating calcium influx through transient potential melastatin 2 (TRPM2) channels, thus decreasing ROS generation in DRG neurons, preventing an accentuation of neuropathic pain, and may be related to its effect on decreasing lipid hydroperoxidation [11,17].

Another culprit in the pain pathway is the dysregulation of glutamate, the primary excitatory neurotransmitter in the central nervous system [18]. NAC has been found to induce the expression of glutamate transporter 1 (GLT-1), the transporter responsible for the largest proportion of glutamate transport and clearance [19,20,21]. In addition, NAC modulates the synaptic release of glutamate by activating the plasma membrane-localized system Xc-, the cystine glutamate transporter, and in doing so, enhancing the endogenous activation of presynaptic mGlu2 receptors [9,21]. This action inhibits glutamate release from excitatory nerve endings and restores extracellular glutamate levels in the nucleus accumbens [9,21]. Overall, NAC helps to maintain glutamate balance to regulate synaptic excitability in the pain-signaling pathway [22,23]. NAC plays a role in the pain pathway by serving as an antioxidant and reducing oxidative stress molecules involved in the pathway, which may explain its ability to reduce neuropathic and musculoskeletal pain, explained further below.

## 5. Analgesic Effect of NAC in Chronic Pain

### 5.1. NAC Mechanism of Neuropathic Pain Reduction

Changes in both the peripheral and central nervous system, whether through disease or injury, can lead to neuropathic pain [24]. Neuropathic pain is estimated to affect 6.9–10% of the population, yet it is difficult to treat due to its unknown mechanism, broad definition, and varying degrees of severity in patients [24,25,26]. NAC has been found to have an analgesic effect in neuropathic chronic pain [21]. Below, we will elaborate on how NAC affects group II metabotropic glutamate receptors and matrix metalloproteinases to help mitigate neuropathic pain.

**Figure 3:**
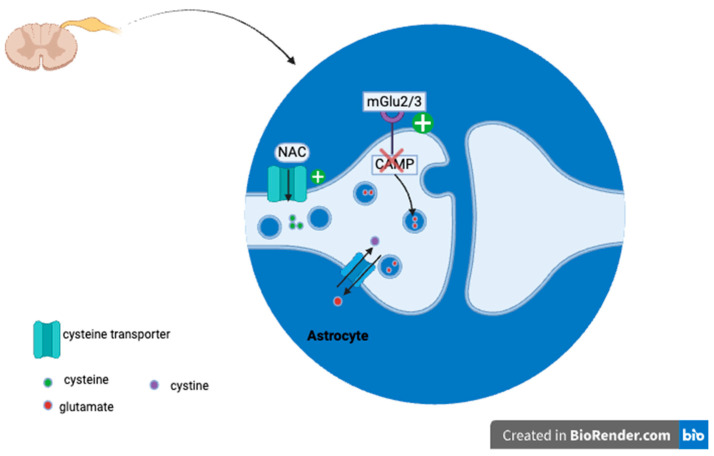
NAC inhibiting peripheral sensitization by ultimately decreasing glutamate release. Through the cysteine transporters, NAC enters the astrocyte, which increases the cystine/glutamate antiport, leading to the intracellular increase of cystine and extracellular release of glutamate. The release of glutamate from astrocytes activates the mGlu2/3 receptor, which inhibits cAMP. This ultimately inhibits the synaptic release of glutamate and reduction of pain.

One of the proposed mechanisms of action of NAC for neuropathic pain relief is via group-II metabotropic glutamate (mGlu) receptors [27]. These receptors are Gαi/o coupled receptors located in the dorsal horn region of the spinal cord [28,29]. NAC is internalized by cysteine transporters, which then increases the concentration of cysteine within the cell. This results in the increased activity of the cystine/glutamate antiporter (Sx_c_^−^), allowing cystine to enter the cell in exchange for glutamate [30,31,32]. The Sx_c_^−^ releases non-synaptic glutamate from astrocytes, leading to the activation of presynaptic mGlu2/3 receptors [33]. These receptors then inhibit cAMP formation and ultimately inhibit the release of synaptic glutamate [34] (Figure 3). 

When mGlu2/3 receptors are activated, neuropathic pain has been shown to be diminished in rat pain models [35,36]. Bernabucci and colleagues found that NAC does not result in analgesia when Sx_c_^−^ is inhibited and in mGlu2 knockout (KO) mice; however, NAC still results in analgesia in mGlu3 KO mice [37]. Truini and coworkers found that pretreating mice with an mGlu2/3 receptor antagonist also prevented NAC’s analgesic effects. Therefore, it is reasonable to conclude NAC results in neuropathic pain reduction via upregulating Sx_c_^−^ which then activates mGlu2/3 receptors [27].

The inhibition of matrix metalloproteinases (MMPs) is another potential mechanism for NAC’s analgesic effect towards neuropathic pain [3]. MMPs are enzymes containing zinc and are converted from pro-MMP to the active enzyme by free radicals [38]. MMP-2 and MMP-9, which are considered to be gelatinases, are believed to play a role in neuropathic pain [38,39]. These MMPs are found in the dorsal root ganglion (DRG). Kawasaki and colleagues found that MMP-9 was upregulated in injured DRG following spinal nerve ligation, while MMP-2 had a delayed response [40]. Both these MMPs produce neuropathic pain via interleukin-1β (IL-1β) cleavage; however, MMP-9 also has early microglial activation and MMP-2 has later astrocyte activation. This makes it probable that MMP-9 is involved in the early phase of neuropathic pain, whereas MMP-2 is involved in the late phase [40]. 

One study showed that when treated with NAC, rats were found to have reduced chronic constrictive injury (CCI)-induced neuropathic pain via NAC inhibiting MMP-2 and MMP-9 [3]. It is likely this inhibition occurred due to NAC’s multiple cysteine residues interfering with the “cysteine switch” needed for MMP activation [3]. The two features of the “cysteine switch” are the pro-peptide domain containing a cysteine residue and the catalytic domain that has a zinc-binding site. The cysteine residue needs to be modified and dissociated from the zinc-binding site to activate MMP-2 and MMP-9 [41,42]. One of the activators of the “cysteine switch” is oxidants and NAC likely has the ability to prevent this residue from being oxidized [3].

**Figure 4:**
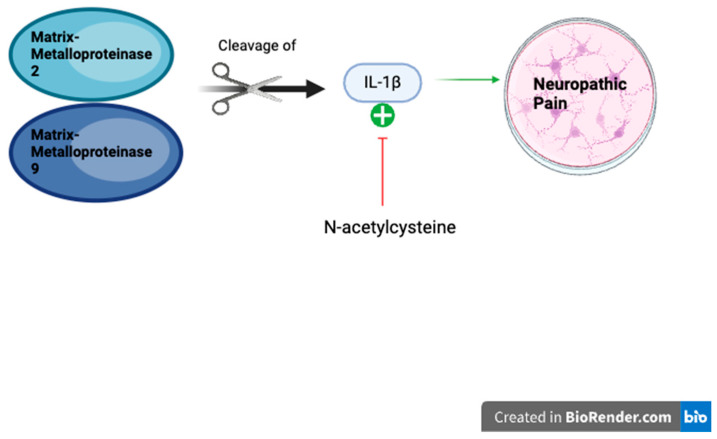
NAC inhibiting mature IL-1β to reduce neuropathic pain. MMP-2 and MMP-9 cleave IL-1β to generate neuropathic pain, which NAC has shown to inhibit in some studies mentioned above. This ultimately decreases neuropathic pain.

NAC has also been found to block the formation and cleavage of mature IL-1β [3,43]. MMP-2 and MMP-9 cleave IL-1β to generate neuropathic pain [40]. Liu and coworkers found that there were increased levels of IL-1β due to MMP-9 in DRGs of incisional rats with intraoperative remifentanil (a narcotic) infusion compared to controls. When treated with NAC, this was attenuated after 2 h [43]. This demonstrates that NAC is involved in the mechanistic pathways of neuropathic pain reduction with respect to matrix metalloproteinases by inhibiting mature IL-1β (Figure 4). If used as an adjuvant, NAC may be able to have a significant therapeutic impact for pain management. 

Recent studies are targeting the NGF/TrkA signaling complex for pain relief. In the presence of inflammation, NGF is released and forms a signaling complex with tropomyosin receptor kinase A (TrkA) on peripheral terminals of nociceptors resulting in hyperalgesia [44]. In a recent phase III trial, tanezumab, a monoclonal antibody antagonizing nerve growth factor (NGF), decreased pain resulting from bone metastases [44]. An in silico and in vitro test showed NAC interferes with this signaling complex by breaking the disulfide bond between cysteine 300 and cysteine 345 in TrkA [45]. Therefore, NGF is not able to activate TrkA, resulting in an analgesic effect.

In summary, it is likely NAC can cause neuropathic pain relief through multiple pathways. NAC can increase Sx_c_^−^ activity and activate presynaptic mGlu2/3 receptors, as well as prevent the “cysteine switch” from being activated by oxidants, leading to the inability to activate MMP-2 and MMP-9, which inhibits the cleavage of IL-1β [3,33,34]. Disrupting the NGF/TrkA signaling complex is another possible mechanism for NAC’s effects [44,45]. Further research for NAC to be used in clinical practice can have optimal effects for patients suffering from chronic pain, as NAC may have the potential to alleviate neuropathic pain.

### 5.2. NAC Mechanism of Musculoskeletal Pain Reduction

Musculoskeletal (MSK) pain occurs in the bones, joints, and tissues of the body [46]. It affects approximately 20% of adults in western civilization. Common conditions causing chronic MSK pain include osteoarthritis (OA), autoimmune inflammatory arthritis, osteoporosis, fibromyalgia, spinal pain, and chronic widespread pain [47,48].

The current literature describes the therapeutic impact NAC may have on MSK pain. Using a rat model, Kaneko et al. found that OA is a result of mechanical stress leading to ROS in chondrocytes which causes chondrocyte apoptosis [49]. Researchers found that NAC can prevent OA and its progression by inhibiting MMP-13 expression, as well as stimulating type II collagen in chondrocytes [50]. In osteoblasts, NO donors and inducible nitric oxide synthase elevate MMP-13 mRNA [51]. Uehara et al. found that oxidative stress was reduced in NAC-treated rats when studying tendon-to-bone healing and saw a significant reduction in MMP-13 expression [52]. MMP-13 is expressed in connective tissue, and its function is to degrade cartilage, including type II collagen [53]. Therefore, by inhibiting MMP-13 expression, it is plausible that NAC can alleviate symptoms, such as musculoskeletal pain caused by OA, since it can prevent its progression and development. However, more research needs to be conducted on NAC’s use because one study by Yeh et al., found that long term NAC use can increase the risk of developing knee OA [54].

Kim et al. found that NAC demonstrated cytoprotective effects in chondrocytes [55]. NAC was able to protect against chondrocyte cytotoxicity by decreasing caspase 3/7 activity and intracellular ROS production, leading to a reduction in chondrocyte apoptosis and necrosis [50]. Xu and colleagues found that when glycine and NAC are given together to rats with spinal cord injuries, there is a protective effect against oxidative stress, delayed atrophy in skeletal muscle, and improved recovery [56]. NAC has also been found to improve the oxidative balance in skeletal muscle and tendon-to-bone healing, as well as muscle strength and resistance to fatigue in skeletal muscle [52,56]. However, Todd et al. found that NAC did not reduce the elevated oxidative stress found in those with ryanodine receptor 1-related myopathies [57]. Therefore, NAC has some therapeutic benefits, but more research needs to be conducted on the targets. It is plausible that NAC is able to reduce oxidative stress by increasing the gene expression of PRDX5, which is an antioxidant enzyme. NAC is also a precursor of glutathione, an antioxidant which is a direct free radical scavenger [52]. Both of these mechanisms regarding PRDX5 and caspase 3/7 activity could contribute to NAC’s ability to prevent and reduce oxidative injury.

### 5.3. NAC Synergistic Patterns with Opioids and/or Acetaminophen

Opioid analgesics have been used to treat acute, cancer, and postsurgical pain [58]. However, their use for chronic non-cancer pain (CNCP) is controversial. A meta-analysis found no significant efficacy differences between opioids and other pharmacological treatments for CNCP [59]. Additionally, long-term use of opioids can lead to side effects such as opioid-induced hyperalgesia (OIH) and analgesic tolerance [60]. Despite these issues, opioids like methadone, tramadol, codeine, fentanyl, and oxycodone are widely used in clinical practice. Opioids function by blocking calcium channels on nociceptive afferent nerves, preventing the release of neurotransmitters like glutamate that contribute to nociception [61]. They also act as NMDA receptor antagonists, with methadone shown to affect NMDA receptors and being used to treat neuropathic pain [61,62]. Few studies have explored the potential synergistic outcomes for pain management from the co-administration of analgesic opioids and NAC which we will discuss below. 

One study was able to show that NAC and morphine have been able to synergistically reduce mechanical allodynia in CPIP rats, which has been attributed to NAC’s antioxidant activity [6]. Although research on the combined activity of NAC and opioids in pain control is limited, extensive studies have demonstrated NAC’s therapeutic efficacy in treating substance use disorders (SUD) by reducing opioid withdrawal and cravings [7,63]. For example, NAC treatment in SUD patients undergoing methadone maintenance therapy decreased depression and anxiety, increased antioxidant capacity and GSH levels, and reduced high-sensitivity C-reactive protein levels compared to the placebo group [64]. NAC also reduced withdrawal behaviors in neonatal rats with neonatal abstinence syndrome, normalizing CNS glutathione and glutamate levels and alleviating acute opioid withdrawal [65]. Chronic consumption of opioids can cause glutamatergic dysregulation in the corticostriatal circuits by decreasing expression of excitatory glial glutamate transporters (GLT1) and catalytic subunit (xCT) of Xc- in the nucleus accumbens (NA). During drug seeking, this decreases glutamate elimination and increases glutamatergic transmission, leading to the increased stimulation of NMDAR and mGluR5, prominent receptors in pain processing and neuropathic pain [66]. However, NAC is able to activate xCT of Xc- system, enabling the exchange of extracellular cysteine with intracellular glutamate. As aforementioned, this increase in non-synaptic glutamate will activate presynaptic mGluR2/3, a common target for analgesic drugs for its ability to regulate threshold pain. mGlu2/3 is an autoreceptor located on glutaminergic terminals of primary afferent fibers in the dorsal horn of the spinal cord; when activated, it inhibits the release of glutamate and, consequently, pain transmission [37]. Preclinical studies on the pharmacological activation of mGluR2/3 have shown how the administration of mGluR2/3 agonists decreases nociceptive behavior in rats and mice [67]. In addition to activating mGlu2/3, increasing non-synaptic glutamate will also increase the expression of xCT and GLT1 and in effect, NAC is able to restore normal glutamatergic transmission and ultimately reduce both pain and cravings. 

NAC has also been shown to reduce postsurgical analgesic opioid consumption. In a randomized prospective pilot trial, NAC administered intraoperatively was shown to reduce postoperative opioid consumption by 16–22% in the first 6–48 h. Patients who were given NAC also reported lower pain scores and took longer to request pain medications, demonstrating the potential synergistic effect of NAC on analgesic opioids [7]. Further research also found that NAC was a potential treatment for opioid-induced testicular dysfunction and degeneration due to its antioxidant properties [68]. These findings are contrary to those of a separate randomized controlled clinical trial that found that those who received pre-emptive administration of NAC after laparoscopic inguinal hernia repair did decrease their postoperative opioid consumption, but there was no significant difference in pain between the NAC and placebo groups. Furthermore, there were higher reports of anaphylactoid reactions in the NAC group. As a result, this study did not recommend using NAC to reduce postoperative pain [69]. 

While limited literature exists on the synergistic patterns between NAC and opioids, there has been significantly more exploration on the therapeutic effects from the co-administration of NAC and acetaminophen, or N-acetyl-para-aminophenol (APAP). APAP is an analgesic and antipyretic used to treat both acute and chronic pain. Numerous studies have explored the use of NAC as the primary treatment for APAP overdose and acetaminophen-induced liver injury. An analysis of a national multicenter study from 1976 to 1985, involving 11,195 cases of acetaminophen overdose, found that NAC was optimally protective in reducing hepatotoxicity within 8 h [70]. NAC prevents hepatotoxicity caused by APAP overdose by providing cysteine to form glutathione (GSH), which detoxifies the electrophilic APAP metabolite N-acetyl-p-benzoquinoneimine (NAPQI). NAPQI depletes GSH stores and increases free radical damage to cells. A previous study also found that increased chemotherapy toxicity, enhanced by high doses of APAP, could be partially reversed with the administration of NAC. NAC was shown to reverse the toxic effects, evidenced by decreased peroxide levels, apoptosis, and mitochondrial damage in tumor cells [71]. This suggests that the administration of NAC in conjunction with APAP has potential to be a therapeutic option that enhances the analgesic effects of APAP by reversing its adverse effects, which is shown by its prevention of liver injury and acetaminophen overdose. 

In addition to treating acetaminophen toxicity, there has been a growing body of literature on other synergistic outcomes from the administration of NAC and APAP. A study found that when NAC and APAP were co-administered in male rats and subsequently given the hot-plate test, NAC was able to dose-dependently augment the analgesic effect of APAP. This study proposes that since NAC is an antioxidant, it can decrease oxidative stress and mitigate central pain transmission, complementing APAP’s ability to inhibit prostaglandin production which can cause chronic pain and inflammation [72]. Another study also showed that APAP and NAC could have a synergistic anti-inflammatory effect. When NAC and APAP were co-administered in piglets, it was able to decrease the levels of inflammatory cytokine IL-10 and NF-κB p65 concentration ratio compared to the control [73]. While there has been an increased number of studies on the use of NAC in conjunction with APAP, there have been very few studies on the use of NAC with other NSAIDs. Some of these studies will be examined in the following sections, The use of NAC to enhance or reverse the toxicity of other non-steroidal analgesics is a topic that has potential for future research and further exploration. 

## 6. NAC Analgesic Effects in Postoperative Pain

NAC is a powerful anti-inflammatory drug that has also been studied for its analgesic effects in postoperative patients. One study examined the effects of NAC in a dose-dependent fashion on pain management following spine surgery [7]. Following the use of high-dose NAC (150 mg/kg) in patients, findings revealed that patients who received NAC required 16–22% less opioid medication within the first two days following surgery compared to those who received a placebo [7]. When adjusted for intravenous NAC intraoperatively, patients were able to reduce their opioid use by up to 22–24%. NAC’s potential analgesic effect is likely linked to its antioxidant properties and ability to inhibit cytokines involved in the inflammatory response, which could explain its effectiveness in reducing acute pain [45]. 

Group-II metabotropic glutamate (mGlu) receptors are activated by N-acetylcysteine (NAC) and have also been described as new potential targets for multimodal pain management. One study investigated whether preoperative administration of NAC (150 mg/kg) in laparoscopic inguinal hernia repairs would lead to a reduction in postoperative pain and opioid consumption [69]. However, no differences in pain reduction were found between the use of NAC and the placebo group. In addition, the study recommended against the use of pre-emptive intravenous NAC due to its high rate of side effects. Ultimately, more studies are needed to evaluate the role of NAC for analgesia. 

Researchers have also conducted recent randomized control studies to show NAC’s role as an adjunct to vitamin C working synergistically to reduce postoperative pain and the effect on opioid consumption after laparoscopic gynecologic oncology surgeries. In a group of 300 patients, all the selected patients were randomly allocated into three groups. Group P was the control group, Group N patients received intravenous injections of NAC (50 mg/kg), and a vitamin C infusion (50 mg/kg) was administered to Group C. Patients were told about the visual analogue pain scale (VAS) preoperatively. In the postoperative period, VAS scores were recorded and noted along with the rescue analgesics received and side effects. Researchers showed that the number of patients who had VAS scores of more than 4 was lower in group C (Vitamin C) at various time intervals when compared to the NAC and placebo groups [27]. It was also statistically significant at 45 min, 60 min, 90 min, 150 min, 180 min, 300 min, and 10 h (*p*-value = 0.014, <0.001, <0.001, <0.001, 0.003, 0.005, 0.006, respectively) [74]. Postoperative opioid consumption was significantly reduced in group C (Vitamin C) compared to the other two groups (*p*-value < 0.001) [74]. This study shows that NAC can be used as a multimodal analgesia, but its specific effect on the reduction of opioid use may be less compared to when paired with vitamin C. If researchers had another group with NAC and Vitamin C infused, there possibly could have also been a reduction in opioid consumption following the procedure, but more research needs to be accomplished on this topic. Overall, intraoperative Vitamin C usage reduced postoperative pain and fentanyl consumption in the postoperative period, and NAC may be used as a part of multimodal analgesia.

## 7. NAC as an Adjunct for Overall Pain Management

Some studies have been able to demonstrate the synergistic effect of NAC and acetaminophen on analgesia and decreasing inflammation [72,73]. NAC has been found to have positive effects on pain when treating patients with neuropathic and painful diabetic neuropathy (PDN). In a study containing 113 patients with Type 2 Diabetes, patients were administered pregabalin, an analgesic used to treat nerve pain, and NAC. Patients administered with both NAC and pregabalin had a greater decrease in mean pain scores than the placebo group. The proposed mechanism was that NAC is able to alleviate the painful system due to its antioxidant properties, suggesting its ability to relieve the oxidative toxic stress and neuroinflammation associated with PDN [75]. 

Findings from numerous studies have also found NAC to be effective as an adjunct in the management of chronic pelvic pain and endometriosis-related pain. A recent study examined the effect of NAC in treating chronic pelvic pain syndrome (CPPS) when co-administered with amitriptyline. Amitriptyline is a medication used to treat depression and nerve pain. This study found that there were significant improvements by week 4 of the study in terms of NIH-CPSI and domain scores. These scores are impacted by pain, urinary symptoms, and quality of life [76]. A prospective cohort study also found that NAC was able to improve endometriosis-related pain and decrease the use of NSAIDs [77]. In support of the use of NAC as an adjunct to NSAIDs in pain management, another study found that NAC was effective when co-administered with Naproxen in treating acute lumbar radiculopathy as a result of intervertebral lumbar disc herniation. However, in contrast with the mentioned studies, a randomized clinical trial found that NAC was not able to significantly improve pelvic pain or the recurrence of endometrioma when given with a low-dose contraceptive after conservative laparoscopic surgery [78]. 

A notable gap in current research exists about NAC as an adjunct to non-steroidal anti-inflammatory drugs (NSAIDs). However, there has been an antinociceptive study that has investigated the analgesic and anti-inflammatory effect of NAC and verapamil in Wistar rats [79]. In this study, the Wistar rats were divided into different groups with the rats being administered saline, diclofenac sodium, NAC, or verapamil. The NAC group was able to prolong hot-plate latency time and decrease paw edema thickness. It was concluded that NAC was able to have an antinociceptive and anti-inflammatory effect similar to that of diclofenac, an NSAID. These results suggest that NAC has potential to be an adjunct to NSAIDs for overall pain management [79].

## 8. Discussion

In this in-depth comprehensive literature review, we demonstrated the various roles of NAC in pain management, including oxidative stress, inflammatory pathways, and synergistic effects with other analgesics. NAC’s capacity in regulating oxidative stress and its impact on neuropathic and musculoskeletal pain has been well-documented [16,55,75,80]. We illustrated studies indicating NAC significantly reduces both neuropathic and MSK pain through mechanisms involving the inhibition of MMPs, modulation of glutamatergic signaling, and direct scavenging of ROS. Additionally, NAC shows promise as an adjunct to opioids and acetaminophen, potentially increasing analgesic effects and reducing opioid consumption.

NAC reduces oxidative stress by boosting GSH levels, which are crucial for cellular defense against oxidative damage [81]. This is important in chronic pain conditions where oxidative stress exacerbates inflammation and pain. NAC reduces pain through the inhibition of NF-kB activation and subsequent production of pro-inflammatory cytokines such as TNF-alpha, IL-1B, and IL-6 [82]. NAC contributes to the regulation of glutamate levels, which in turn helps modulate synaptic excitability within the pain signaling pathway. By acting as an antioxidant and diminishing the impact of oxidative stress molecules associated with this pathway, NAC may provide an explanation for its effectiveness in alleviating neuropathic and musculoskeletal pain. Moreover, NAC may effectively alleviate neuropathic pain by activating group-II metabotropic glutamate receptors and inhibiting matrix metalloproteinases. By enhancing the cystine/glutamate antiporter’s activity and disrupting inflammatory pathways, NAC shows promise as an adjunct therapy in managing neuropathic pain. Meanwhile, NAC also demonstrates potential therapeutic effects in alleviating musculoskeletal pain, particularly through its ability to inhibit MMP-13 expression and enhance type II collagen production in chondrocytes. However, further research is needed to clarify its long-term safety and efficacy, notably in managing musculoskeletal disorders and other neuropathic disorders, especially considering some inconclusive findings. The NGF/TrkA signaling complex demonstrates another aspect of NAC’s potential in pain relief. NAC’s ability to disrupt disulfide bonds between key cysteine residues in TrkA inhibits NGF from activating this receptor, reducing hyperalgesia. Given the role of NGF/TrkA in inflammatory pain, this mechanism presents a promising therapeutic target.

The synergistic patterns of NAC with opioids and acetaminophen have potential in enhancing pain relief. Studies have demonstrated that NAC can enhance the effects of opioids. A study by Hodebourg et al. found that NAC significantly reduced opioid self-administration in a rat model of opioid use disorder after chronic exposure to opioids [83]. This indicates NAC’s potential in enhancing opioid therapy by possibly reducing the required dose and reducing dependency. While research on NAC–opioid synergy is limited, NAC’s role in treating acetaminophen toxicity highlights its ability to reduce oxidative stress and boost pain relief. More studies are needed to further explore these synergistic effects.

NAC’s ability to reduce opioid consumption postsurgery and its role in reducing opioid withdrawal symptoms in substance use disorders further demonstrates its potential benefits. In a previous study on the effects of NAC on opioid consumption, a treatment group that was administered NAC, versus the control group, tended to consume less morphine within the first 48 h after knee ligamentoplasty [80]. On the other hand, some studies have shown that NAC may increase the risk for developing knee osteoarthritis [54,83]. One study showed that NAC was associated with a four-fold increased risk of knee osteoarthritis in patients that received oral NAC over 28 days within 1 year compared to the control group that did not receive NAC. Additional research on the safety and efficacy of NAC is necessary before incorporating it in routine management.

## 9. Future Perspectives

The incorporation of NAC into chronic pain management may be beneficial in reducing the reliance on opioids and improving patient outcomes. Its potential to lower opioid doses required for pain relief can reduce the risks associated with long-term opioid use, such as tolerance and dependence. Moreover, the ability of NAC to enhance the efficacy of acetaminophen provides an additional non-opioid option for pain management [72]. It would be beneficial if future research on NAC would focus on exploring optimal dosing regimens and evaluating long-term outcomes and side effects in diverse patient populations. It would also be worthwhile to study the interactions between NAC and cannabinoids and their effect on pain management, as there is very limited research on this topic. Conducting further research on NAC would be useful and even life-changing if it can be shown to be used in clinical medicine and potentially improve the quality of life of patients experiencing chronic pain.

## 10. Conclusions

In conclusion, NAC, serving as an antioxidant and precursor to GSH, does show some therapeutic benefit for chronic pain management when used both in isolation and as a promising adjunct. Its role in reducing ROS and inhibiting the matrix metalloproteinases is valuable insight in understanding its mechanism. More research needs to be conducted to illustrate specific targets of where NAC acts to reduce both MSK and neuropathic pain. NAC’s mechanisms of action, including the inhibition of matrix metalloproteinases and modulation of glutamate pathways, contribute to its analgesic effects. Moreover, NAC demonstrates potential synergistic benefits when combined with opioids and acetaminophen, potentially reducing opioid consumption and enhancing pain relief. Despite its advantages, further research is essential to fully understand NAC’s efficacy, optimal dosing, and long-term effects in diverse pain management scenarios. If accomplished, NAC could have beneficial long-term impacts on patients suffering from debilitating chronic pain, improving the quality of life for patients, communities, and beyond. 

## Data Availability

PubMed, Google Scholar, NHI.
